# A Comparative Study on the Performance and Microstructure of 304NG Stainless Steel in Underwater and Air Laser Welding

**DOI:** 10.3390/ma17153854

**Published:** 2024-08-03

**Authors:** Jiaqi Sun, Yue Yang, Kai Wang, Shaohua Yin, Zhen Li, Zhen Luo

**Affiliations:** 1 School of Materials Science and Engineering, Tianjin University, Tianjin 300350, China; 2 China Nuclear Power Operations Co., Ltd., Shenzhen 518124, China; 3 Suzhou Nuclear Power Research Institute Co., Ltd., Suzhou 215004, China

**Keywords:** underwater laser welding, stainless steel, welding morphology, microstructure, mechanical properties

## Abstract

In order to facilitate the application of underwater laser welding technology in in situ repairs of nuclear power plants, this study conducted comparative experiments between local dry underwater laser welding and laser welding in air on 304NG nitrogen-controlled stainless steel. The aim was to explore its microstructural evolution and mechanical properties in underwater environments. It was found that, near the fusion line of laser welding in air, columnar dendrites gradually evolved into cellular dendrites toward the weld center, eventually disappearing, resulting in a skeletal ferrite and serrated austenite structure. The underwater laser welding joints exhibited similar characteristics yet with more pronounced alternation between columnar and cellular dendrites. Additionally, the size of cellular dendrites decreased significantly, and needle-like ferrite was observed at the weld center. The hardness of underwater laser welded joints was slightly higher than that of in-air laser welded joints. Compared to laser welding in air, the strength of underwater laser welding joints increased from 443 MPa to 471 MPa, and the displacement increased from 2.95 mm to 3.45 mm, both types of welded joints exhibited a mixed mode fracture characterized by plasticity and brittleness.

## 1. Introduction

Nitrogen-containing austenitic stainless steel uses nitrogen as a strengthening element, achieves low carbonization through secondary refining technology, and improves corrosion resistance, becoming a mainstream material in nuclear power plant projects [1,2]. However, in actual applications, as nuclear power plant equipment ages, underwater nuclear reactor equipment may suffer various damages when operating in high-radioactivity and high-temperature environments. To ensure the safe operation of nuclear power plants, immediate in situ remediation of underwater nuclear reactor equipment has become an important challenge that needs to be solved urgently [3]. Currently, underwater welding, especially underwater arc welding, is widely used when performing in situ repairs of nuclear power plants at home and abroad [4]. However, underwater arc welding faces numerous limitations, being greatly affected by factors such as water depth, water quality, and water flow. It tends to form larger heat-affected zones and generates slag and waste materials. Moreover, the welding process exhibits poor stability and poses high safety risks [5]. Laser beam welding, as a highly precise and efficient welding method, holds great potential for widespread applications. It features a small heat-affected zone, non-contact operation, and ease of integration with automated systems. These characteristics help reduce deformation, maintain the original properties of materials, minimize pollution and oxidation risks, and enhance production efficiency [6]. These characteristics endow underwater laser beam welding technology with unique advantages in the remote and precise repair of nuclear power equipment, particularly in the maintenance and repair of core components of reactors.

The research on underwater laser beam welding primarily focuses on two aspects: wet underwater laser beam welding and local dry underwater laser beam welding. Guo et al. [7] investigated the impact of water depth on underwater laser beam welding using wet underwater laser beam welding technology. The research findings indicate that, under the influence of the incident laser, complex interactions occur among the laser, water, and material, forming a “beam channel”. When the water depth is less than 3 mm, the effect of water on the “beam channel” is weak. However, when the water depth is greater than 7 mm, the effect of water significantly increases, potentially leading to a welding failure. You et al. [8] employed specific flux-assisted wet underwater laser beam welding technology and investigated the effect of water depth on duplex stainless-steel welded joints. The research findings demonstrate that, even in 15 m water depth, ideal welding joints could still be obtained. Furthermore, the weld penetration depth under deepwater conditions was consistently smaller than that under shallow water conditions, and increasing heat input did not significantly increase the penetration depth. Huang et al. [9] utilized digital image correlation technology to investigate the bending behavior of underwater laser welded 304 stainless-steel thin plates. The research findings reveal that the arch-shaped deformation of the weld is caused by the heterogeneous temperature gradient of the plate during the welding process. The deformation increases with the increasing heat input. In addition to heat input, the width of the weld seam also significantly influences the deformation of underwater laser beam welding. A wider weld seam results in a greater degree of bending deformation. Guo et al. [10] employed local dry underwater laser beam welding technology to systematically study the effects of different welding process parameters on underwater laser butt joints. Experimental results demonstrate that, with the increase of heat input, the porosity of the butt joints initially decreases and then increases. The optimal butt joint was obtained when the laser power was 2 kW and the welding speed was 1.0 m/min. At this point, the tensile strength, impact toughness, and microhardness of the joints are close to the performance level of laser-welded joints in air. The optimization of process parameters has been extensively studied. However, the reliability of welded structures ultimately determines the engineering safety and economic benefits. Theoretically, under good drainage conditions, an environment similar to that in the air can be formed. Moreover, forced cooling technology using water as the cooling medium is generally considered to be an effective means of grain refinement [11,12]. Consequently, theoretically, the performance of underwater laser beam welding joints can exceed that of joints in air. Unfortunately, the current research reports indicate that the performance of underwater welding joints is inferior to those in air environments. Therefore, optimizing underwater laser beam welding systems and improving the quality of welding joints are of significant importance. In addition, the drainage effect may be an important factor affecting the welding quality. We optimized the drainage cover according to the drainage effect.

This study conducted local dry underwater laser beam welding on 304NG nitrogen-containing stainless steel using an optimized double-layer air curtain drainage cover and performed a comparative analysis with laser beam welding in air. The aim was to explore the microstructure evolution and mechanical properties of 304NG nitrogen-containing stainless steel during the underwater laser beam welding process. At the same time, the underwater laser welding system was optimized, reducing engineering safety risks and saving manpower and material resources.

## 2. Materials and Methods

**Figure 1:**
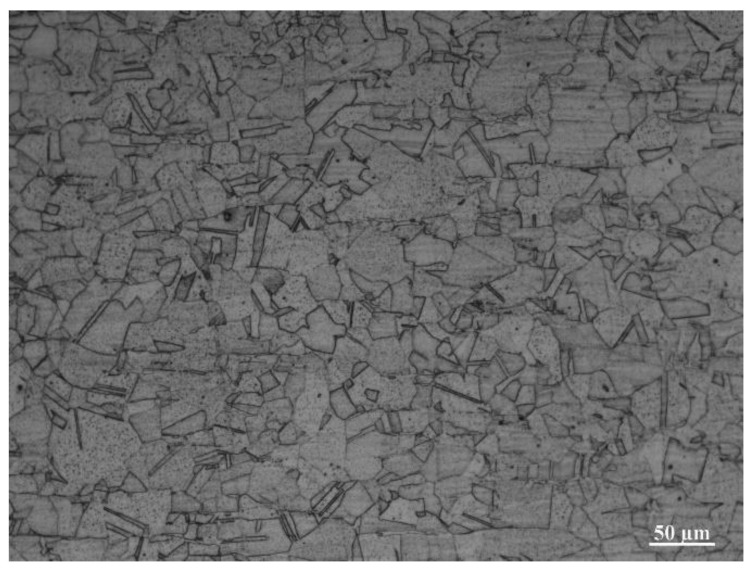
The microstructure of 304NG nitrogen-containing stainless steel.

**Figure 2:**
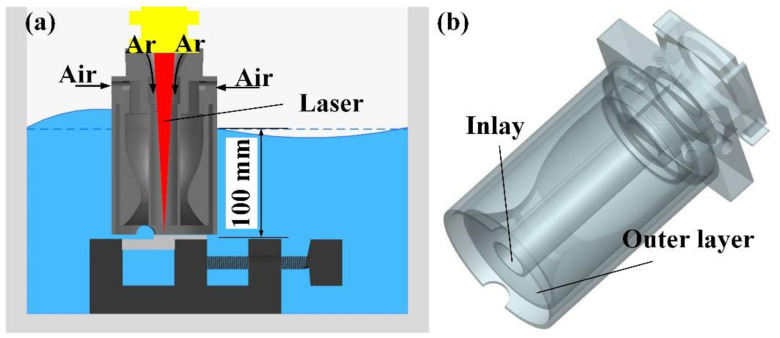
(**a**) Schematic diagram of underwater laser beam welding and (**b**) 3D structural of drain cover.

The 304NG stainless sheets (Mingxing Metal Materials Co., Ltd., Dongguan, China) with a dimension of 100 mm × 50 mm × 5 mm and 100 mm × 50 mm × 2 mm were selected as the base materials in this study. The chemical compositions of the 304 stainless-steel sheets are listed in Table 1; their microstructure is shown in Figure 1. Two experimental environments were studied: in air and underwater. The sheets were all overlap-welded and the overlap length was 20 mm. The local dry underwater laser beam welding experiments were performed using a dual-layer gas curtain drainage cover, as illustrated in Figure 2. The HL-CM-10000 fiber laser (Daqo Laser Science and Technology Industry Group Co., Ltd., Shenzhen, China), capable of reaching a maximum power output of 10 kW and operating at a wavelength of 1060 nm, was utilized. Prior to welding, the surface of the plates was sanded with sandpaper, polished, and cleaned with alcohol to eliminate surface oil and impurities. The welding parameters of the in-air and underwater environments included laser power of 3.5 kW, welding speed of 8 mm/s, air as drainage gas with gas flow rate of 80 L/min, Ar with a purity of 99.9% as shielding gas with gas flow rate of 30 L/min. In the underwater environment, the water depth was 100 mm.

**Figure 3:**
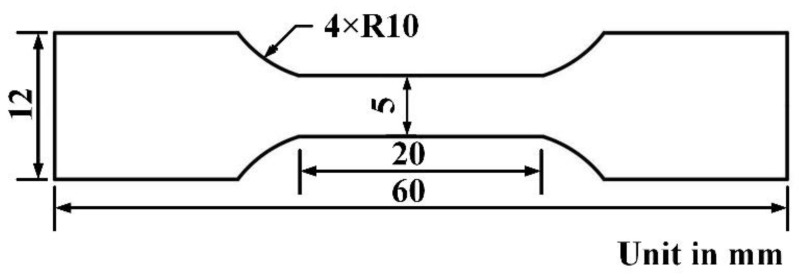
The type of specimens for tensile testing.

Metallographic etching was performed using aqua regia with a HNO_3_: HCl ratio of 1:3. The macrostructure of the lap joints was observed using an ultra-deep-field microscope (UDM, Smartzoom5, Carl Zeiss AG, Oberkochen, Germany), while the microstructure was observed using an optical microscope (OM, ZEISS, Carl Zeiss AG, Oberkochen, Germany). The Vickers microhardness testing (HUAYIN HV-1000A, Huayin Testing Instrument Co., Ltd., Laizhou, China) was conducted with a preload of 200 g applied and maintained for 15 s. Tensile shear tests were carried out at room temperature on a universal testing machine (UTM6104, Sansi Zongheng Technology Co., Ltd., Shenzhen, China) at a testing speed of 2 mm/min. The tensile shear specimens were dog-bone standard tensile samples with a length of 60 mm and a cross-sectional width of 5 mm, as shown in Figure 3. Finally, the fracture surfaces of the tensile shear specimens were observed using a scanning electron microscope (SEM, JSM 7800F, JEOL Ltd., Tokyo, Japan).

## 3. Results

### 3.1. Macromorphology

**Figure 4:**
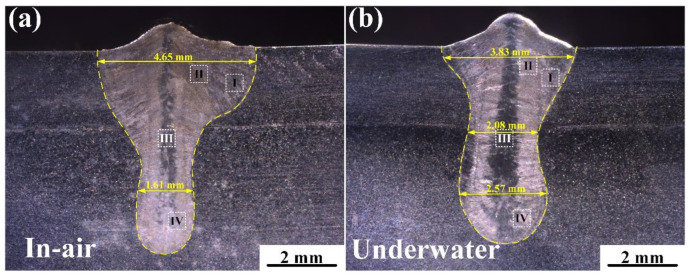
Macromorphology of the overlap joints under different welding conditions: (**a**) in air and (**b**) underwater.

Figure 4 shows the macroscopic morphology of the laser-welded joints in air and underwater. It was found that both joints exhibited no obvious porosity, cracks, or other defects, demonstrating good formation quality for the in-air and underwater environments. The in-air welded joint had a broad top and a narrow bottom, whereas the underwater welded joint exhibited a characteristic of being wider at the top and bottom and narrower in the middle. Additionally, the penetration depth and frontal width of the in-air welded joint were greater than those of the underwater welded joint.

### 3.2. Microstructure

**Figure 5:**
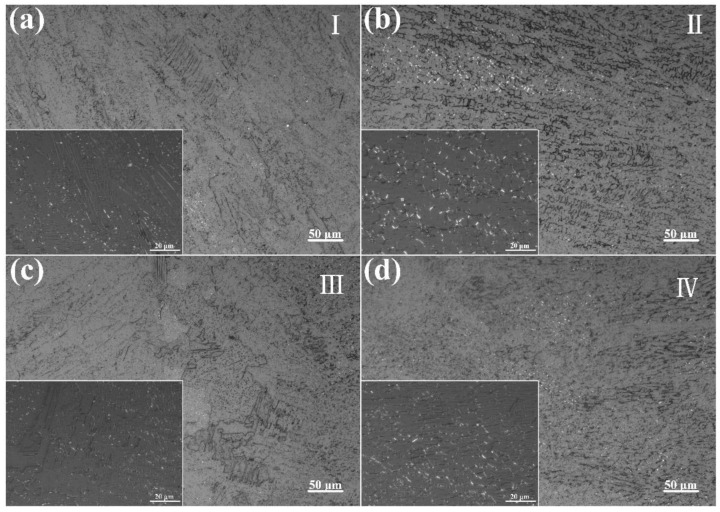
Microstructure of the in-air welded joints in Figure 4a: (**a**) region I, (**b**) region II, (**c**) region III and (**d**) region IV.

**Figure 6:**
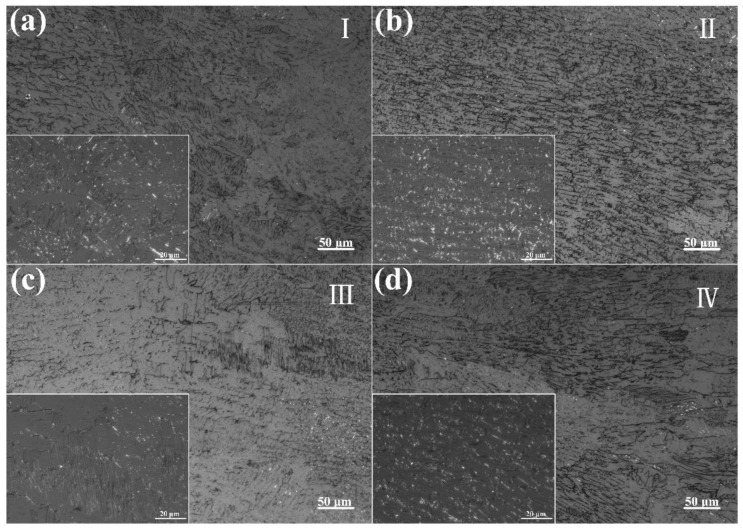
Microstructure of the underwater welded joints in Figure 4b: (**a**) region I, (**b**) region II, (**c**) region III and (**d**) region IV.

The microstructure of the fusion zone was examined using a metallographic microscope, as shown in Figure 5 and Figure 6. Each joint was divided into four different regions. In Region I of the in-air welded joint, relatively orderly columnar dendrites growing toward the weld center were observed. As the weld center approached, these columnar dendrites were gradually replaced by cellular dendrites. Near the weld center, the cellular dendrites disappeared, revealing a skeletal ferrite structure and a serrated austenite structure, with columnar-like morphologies growing toward the weld center.

The underwater welded joint’s microstructure was comparable to that of the in-air welded joint. However, it is worth noting that columnar and cellular dendrites alternated in Region I of the underwater welded joint, attributed to the smaller columnar dendrite region in the underwater welded joint compared to that in the in-air welded joint. Additionally, the cellular dendrites in Region II of the underwater welded joint were significantly smaller than those in the in-air welded joint [13]. Conversely, near the weld center, the cellular dendrites disappeared, and both skeletal ferrite and acicular ferrite structures were observed. In contrast, only skeletal ferrite was found in the in-air welded joint.

### 3.3. Mechanical Properties

**Figure 7:**
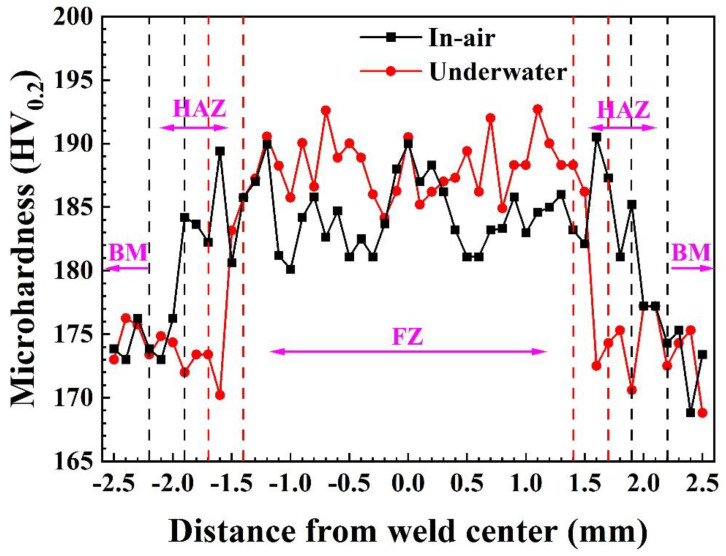
Microhardness distribution of the welded joints.

The microhardness distribution of the welding joints using underwater laser and laser in air is shown in Figure 7. The microhardness lowered significantly from the fusion zone to the base material. This is because the weld fusion zone has a larger number of equiaxed grains, and the higher grain boundary density poses a greater obstacle to dislocation movement [14,15]. Additionally, compared to an in-air welded joint, an underwater welded joint has a slightly higher microhardness. This is primarily due to the underwater laser welding process, where water acts as a cooling medium, effectively refining the grain structure and increasing the number of grain boundaries, thus enhancing the material’s hardness.

**Figure 8:**
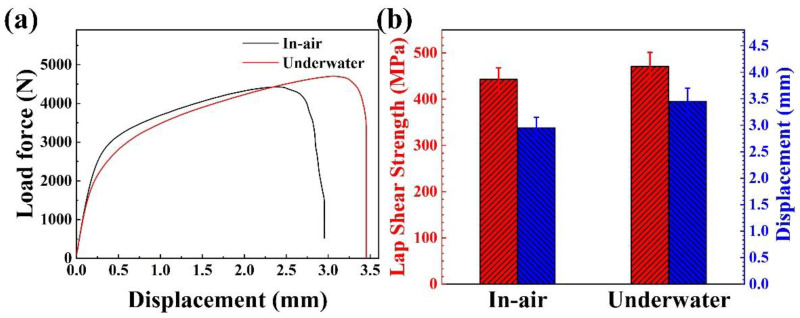
(**a**) Displacement–force curve and (**b**) corresponding lap shear strength and displacement.

Figure 8 presents the tensile characteristics of the two joints, indicating that a plastic fracture occurred [16,17]. The underwater welded joint exhibited higher strength and plasticity. Compared to the in-air welded joint, the strength of the underwater welded joint increased from 443 MPa to 471 MPa, and the displacement increased from 2.95 mm to 3.45 mm. 

**Figure 9:**
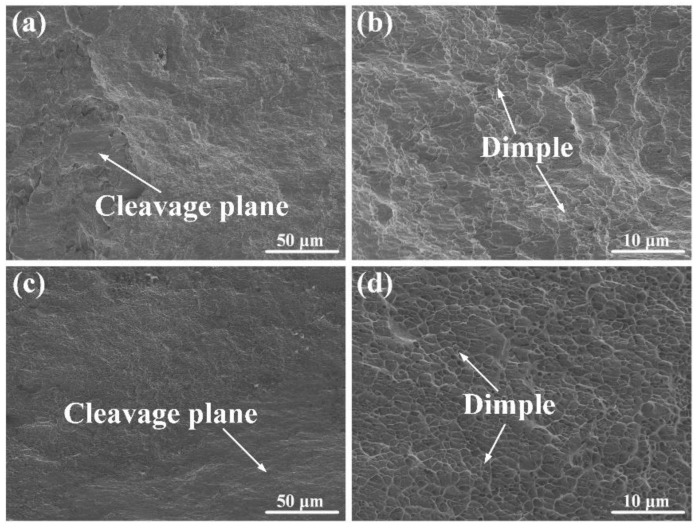
SEM of fracture surface morphology of joints: (**a**,**b**) in air and (**c**,**d**) underwater.

To gain a deeper understanding of the fracture mechanism, the fracture surface morphologies of the two joints were examined with an SEM, as illustrated in Figure 9. The observations revealed that both joints exhibited dimples and cleavage planes on the fracture surfaces. However, the underwater welded joint had a larger dimple density than the in-air welded joint.

## 4. Discussion

In underwater environments, laser beams transmit through the water medium to the welding area. Water exhibits absorption and scattering effects on the laser, which can even lead to a direct failure of laser welding. The drainage cover isolates the welding area from water and uses protective gas to expel water and oxygen, providing a stable welding environment. Inside the drainage cover, the dry environment allows the laser beam to directly impact the metal surface without interference from water. The laser beam focuses on the surface of the 304 stainless-steel sheet, with the energy being absorbed by the material and converted into heat, rapidly melting the material to form a molten pool [18]. The protective gas inside the drainage cover (in this case, argon) expels water and oxygen, preventing oxidation of the metal during welding and maintaining the purity of the molten pool. The molten pool remains stable and moves along with the laser beam. The dry environment and protective gas within the drainage cover ensure that the formation and dynamic behavior of the molten pool are similar to those in air-based laser welding.

During laser welding, metal vapor and keyholes are generated on the surface of the nitrogen-controlled stainless steel under the irradiation of the high-energy laser beam. The keyhole remains stable under the action of a recoil force, balanced with gravity and surface tension. The recoil force is affected by the effective intensity of the beam as it interacts with the keyhole wall, which is due to the distribution of laser beam intensity and refraction frequency within the keyhole walls [19]. The liquid flow outside the keyhole walls and the surface tension correspond to the continuously generated vapor pressure within the keyhole cavity, maintaining a dynamic balance [20]. The combined effects of surface tension, recoil force, and gravity determine the liquid metal flow pattern. Typically, the Marangoni effect usually happens in the top part of the weld, where Marangoni convection causes the liquid metal to move from the keyhole’s center to the periphery. The surface tension in the molten pool enhances this effect, resulting in a significantly greater width in the upper area of the weld than in the middle and lower areas [21]. In underwater environments, the molten pool cools faster because the surrounding water has a cooling impact, which weakens the Marangoni effect [22]. However, despite this, the upper portion of the weld’s breadth is wider than its middle and bottom halves. For lap welding, due to the possible presence of some water between the two plates, which increases the cooling rate of the middle part of the weld, the middle portion of the weld’s width is less than that of the upper and lower parts of the weld.

During the welding process, the laser beam rapidly heats the material surface, forming a high-temperature molten pool. Due to the laser beam’s cooling speeds, the liquid metal in the molten pool solidifies quickly. The welding thermal cycle and the cooling rate determine the type and morphology of the microstructure [23,24]. At the edges of the molten pool, where the cooling rate is highest, grains grow rapidly along the temperature gradient, forming columnar dendritic structures. In an underwater environment, the cooling rate increases further, preventing the columnar dendritic structures from fully developing, resulting in a smaller columnar dendritic region in the weld compared to that in air [25]. Closer to the center of the molten pool, the cooling rate decreases, though it remains relatively high, allowing dendrites to penetrate deeper into the liquid. Concurrently, the lateral sections of the dendrites experience constitutional supercooling, leading to the formation of short secondary arms [26]. However, due to the small spacing between the primary trunks, the secondary arms remain short, resulting in a distinctive cellular dendritic structure [27]. The underwater environment’s higher cooling rate further limits the growth of secondary arms, yielding finer cellular dendritic structures. In the center of the molten pool, where the temperature is highest and the cooling rate is slower, ferrite is allowed to grow along the temperature gradient at high temperatures, forming a skeletal structure [28]. In the underwater environment, the significantly increased cooling rate leads to the rapid nucleation and growth of ferrite at lower temperatures, forming fine acicular ferrite.

In general, the mechanical properties of materials are influenced by their phase composition, grain size, and microstructural morphology [29]. For laser-welded joints in in-air and underwater environments, the phase composition remains consistent. Under these circumstances, the size and morphology of the microstructure become the primary factors affecting the material properties. The Hall–Petch relationship describes the correlation between a material’s yield strength and grain size [30]:

(1) $$\sigma_{s}=\sigma_{0}+kd^{-\frac{1}{2}}$$

where *σ_s_* is the yield strength, *σ*_0_ is the intrinsic stress of the material, *k* is the Hall–Petch coefficient, and *d* denotes the grain diameter. The yield strength of a material increases as the grain size decreases. This is due to the enhanced hindrance effect of grain boundaries on dislocation movement, making it more difficult for dislocations to move within smaller grains, thereby increasing the material’s strength. Grain refinement can improve the uniformity of plastic deformation, reducing local stress concentration, and thus enhancing the overall plastic deformation capacity of the material [31]. Consequently, the strength and ductility of underwater laser welded joints are superior to those of joints welded in air.

## 5. Conclusions

In this study, 304NG stainless steels were welded using in-air laser welding and partial dry underwater laser welding. The objective was to investigate the welding mechanism, microstructural development, and mechanical properties of 304NG nitrogen-controlled stainless steel during in-air and underwater laser welding. The following conclusions were drawn:

1. The macroscopic morphology of the laser weld joint in air exhibited a wider upper part and a narrower lower part, whereas the underwater laser weld joint displayed a wider upper and lower part with a narrower middle section. Both joints exhibited no obvious porosity, cracks, or other defects.
2. The weld joints produced by both welding methods showed a transition from columnar dendrites at the weld edge to cellular dendrites and equiaxed grains toward the weld center. Compared to welding in air, the columnar dendrite zone and equiaxed grain zone in underwater laser welding were smaller, while the proportion of the cellular dendrite zone was larger. Needle-like ferrite was also observed in the center of the weld seam for underwater laser welding. Additionally, the grain size of the joints obtained by underwater laser welding was finer.
3. The hardness, strength, and plasticity of joints welded underwater were slightly higher than those of joints welded in-air. The fracture mode of joints produced by both welding methods was a ductile fracture. Compared to welding in air, the tensile strength of the underwater-laser-welded joints increased from 443 MPa to 471 MPa, and the displacement increased from 2.95 mm to 3.45 mm.

## Figures and Tables

**Table 1:** The chemical composition of 304NG nitrogen-containing stainless steel.

Element	C	Si	Mn	P	S	Cr	Ni	N	Cu	Fe
Wt.%	0.035	0.98	2.00	0.03	0.0015	18.90	9.48	0.06	0.95	Bal.

## Data Availability

The original contributions presented in the study are included in the article, further inquiries can be directed to the corresponding authors.
